# A Ten-Year-Old Boy with Antiepileptic Drugs-Induced DRESS Syndrome

**DOI:** 10.1155/2020/8837607

**Published:** 2020-09-11

**Authors:** S. Vithana, M. H. A. D. De Silva, G. P. Hewawitharana

**Affiliations:** ^1^Lady Ridgeway Hospital for Children, Colombo, Sri Lanka; ^2^Deparment of Paedaitrics, Faculty of Medicine, University of Ruhuna, Galle, Sri Lanka; ^3^Teaching Hospital Karapitiya, Galle, Sri Lanka

## Abstract

Drug reaction with eosinophilia and systemic symptoms (DRESS) syndrome is a life-threatening adverse drug reaction if it is not timely diagnosed and treated. This happens probably following a cascade of immune reactions after the administration of the drug ultimately leading to multiorgan failure and death. Several groups of drugs have been identified as potential aetiologies but the commonest one identified is antiepileptic drugs. The clinical features of DRESS syndrome usually appear several weeks after commencing the offending drug. Initially, fever lymphadenopathy and rash appear followed by hepatitis. Rash is the most prominent feature, and it is a generalized erythematous nonblanching maculopapular rash without the involvement of the mucus membranes or eyes. The rash desquamated over the following days and changed it's context to an exfoliative dermatitis. We report a case of a 10-year-old boy who is one of the twins born to nonconsanguineous parents at 34 weeks of gestation.

## 1. Introduction

Drug reaction with eosinophilia and systemic symptoms (DRESS) syndrome, also referred to as drug-induced hypersensitivity syndrome (DIHS), is a distinct and a potentially life-threatening adverse reaction caused by certain drugs. They present with fever, rash, lymphadenopathy, hematologic abnormalities, and manifestations of multiorgan failure. Anticonvulsants and sulfonamides are the most common offending agents. DRESS syndrome may have significant multisystem involvement, including hematological, hepatic, renal, pulmonary, cardiac, neurologic, gastrointestinal, and endocrine abnormalities. It has a mortality rate of about 10%, which occurs commonly due to fulminant hepatitis with hepatic necrosis.

## 2. Case Presentation

A 10-year-old boy, an offspring of nonconsanguineous parents, presented with a generalized rash which appeared on day 3 of fever. Temperature was more than 103°F (39.4°C), but there was no obvious focus of an infection. He was the second-born twin at 34 weeks of gestation. His neonatal period was complicated with meningitis and convulsions, and he was on sodium valproate (20 mg/kg/day) since then. Two months prior to the current presentation, a follow-up EEG suggested that there were only focal changes; hence, he was commenced on carbamazepine (20 mg/kg/day), and sodium valproate was tailed off.

**Figure 1:**
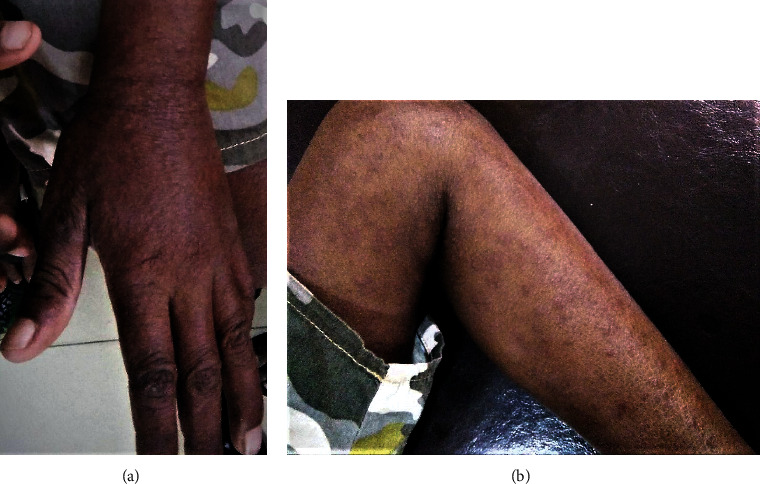
Exfoliative dermatitis in the upper limb (a) and the lower limb (b) which was initially presented as an erythematous nonblanching maculopapular rash.

On examination, he was febrile and was having a generalized erythematous nonblanching maculopapular rash without involvement of the mucus membranes or eyes. The rash desquamated over the following four days and changed it's context to an exfoliative dermatitis (Figures 1(a) and 1(b)). There was bilateral cervical lymphadenopathy of 2 cm in size. Examination of the respiratory system revealed bilateral pleural effusions without any added sounds on auscultation. Hepatomegaly was noted, and the liver was extending 4 cm below the costal margin with moderate ascites.

The total white blood count was 15.4 × 10^3^/mm^3^ (normal from 4.0 to 10.0 × 10^3^/mm^3^) with 38% neutrophils, 31% leukocytes, and 16% eosinophils (absolute count: 2.5 × 10^3^/mm [3]). Hemoglobin concentration was 13.2 g/dl, and the platelet count was 140 × 10^3^/mm [3]. Normochromic normocytic red blood cells with moderate eosinophilia and mild thrombocytopenia was detected on the blood picture. There were no abnormal or immature cells. ESR was 07 mm in the 1^st^ hour. Serum bilirubin was 6.8 mg/dl (0.3–1.0 mg/dl). SGOT and SGPT were 810 U/L (normal: from 0 to 37 U/L) and 735 U/L (normal: from 0 to 41 U/L), respectively. Serum total protein and serum albumin were 55 g/L and 33 g/L, respectively, and PT/INR was 1.2. His renal functions were normal. Ultrasound scan of the abdomen and chest revealed hepatomegaly, bilateral moderate amount of pleural effusion, and ascites. Chest X-ray confirmed the bilateral pleural effusions. Paracetamol levels were below detectable levels. Hepatitis screening was negative for hepatitis A, B, and C. Electrocardiogram (ECG) and echocardiogram were both normal. EBV, CMV testing, and HIV screening test were all negative. Antinuclear antibodies (ANA) and double-stranded DNA (DSDNA) were also negative.

Carbamazepine was omitted, and levetiracetam of 20 mg/kg/day was commenced to control seizures. Pyridoxine was not used simultaneously as this was a relatively low dose and later due to the absence of any behavioral manifestations. After commencement of prednisolone 2 mg/kg/day, he responded completely within two weeks of therapy. This was tailed off over a period of one month. He experienced no flare after corticosteroid tapering or withdrawal and has not had any hepatic sequelae. Follow-up at twelve months after discharge revealed that the patient had no recurrence of his rash or other symptoms, seizures were well controlled, and there was normalization of his serum transaminases. Levetiracetam was continued at the same dose, and there was no adverse effects including behavioral changes during the follow-up of this patient.

## 3. Discussion

DRESS syndrome was first recognized in 1950 by Chaiken, in a patient who was using anticonvulsants. Later, patients with DRESS syndrome were found to have started on one of the few selected medications during the last two to eight weeks (Table 1) with antiepileptics being the most commonly implicated. Vitamin D deficiency has been associated to the pathogenesis of DRESS due to its protective action against inflammatory and autoimmune conditions.

Clinically, this syndrome includes an extensive mucocutaneous rash, fever, lymphadenopathy, hepatitis, hematologic abnormalities with eosinophilia, and atypical lymphocytes. And, it may involve other organs with eosinophilic infiltration, producing damage to vital organs, especially the kidneys, heart, lungs, and pancreas [1].

This multivisceral involvement differentiates DRESS from other common skin reactions to drugs. Another unique feature of this syndrome is its late onset in relation to the period of introduction of the causative drug. The reactions occur around 3 weeks to 3 months after initiating the offending drug [2, 3]. The other feature is the persistence or worsening of the symptoms despite the withdrawal of the offending drug. The exact mechanism of DRESS remains unclear but, in cases related to anticonvulsants drugs, three possible aetiologies are considered: (i) deficiency or abnormality of the epoxide hydroxylase enzyme that detoxifies the metabolites of aromatic amine anticonvulsants (metabolic pathway); (ii) associated sequential reactivation of herpesvirus family; and (iii) ethnic predisposition with certain human leukocyte antigen (HLA) alleles (immune response) [4].

Typically about 2 months after commencing the offending agent, the patients will present with fever (38°C–40ºC), which is the most common symptom, and rash [5]. The cutaneous eruption consists of a morbilliform rash, which is difficult to differentiate with other less-severe drug reactions [6]. The face, upper trunk, and upper extremities are initially affected with subsequent progression to the lower extremities [7].

Lymphadenopathy is the commonest visceral manifestation, and it could be limited to one group of lymph nodes or it may be a generalized lymphadenopathy. Lymph nodes become painful, and they gradually resolve with the withdrawal of the offending drug [5]. Various hematologic abnormalities are also observed in DRESS syndrome. These consist of marked leukocytosis, eosinophilia, and atypical lymphocytes. These findings guide the diagnosis towards DRESS syndrome; however, it could be difficult to differentiate from viral infections such as Epstein–Barr virus or other hematologic diseases with mononucleosis [5]. Multiorgan involvement may lead to myocarditis, pericarditis, interstitial nephritis, necrotizing granulomatous vasculitis of the kidney, encephalitis or meningitis, colitis, and thyroiditis [8, 9]. Arthritis or arthralgia and myositis may also occur in patients with DRESS syndrome [8]. Hepatic involvement with significant hepatomegaly is the second most common visceral manifestation. Hepatitis with isolated elevation of liver transaminases is common. The patients are usually anicteric, but fulminant hepatic failure is the main cause of death due to DRESS syndrome [8]. Widespread or focal hepatic necrosis is seen in liver biopsies in these patients [10]. Renal involvement is also recognized. There is usually an increase in serum creatinine and urea and a decrease in creatinine clearance. In urine, increased content of eosinophils can be observed [9]. Neurological complications include aseptic meningitis and encephalitis [9]. The exclusion of other serious infections, neoplastic diseases, and autoimmune or connective tissue disorders is necessary for an accurate diagnosis of DRESS syndrome. Complications are rare and include limbic encephalitis, thyroid disease, renal failure, splenic rupture, eosinophilic colitis, eosinophilic esophagitis, enterocolitis, and fatal CMV [11].

The early recognition of adverse drug reaction and withdrawal of the offending drug are the most important and essential steps towards the clinical improvement. Empirical treatment with antibiotics or anti-inflammatory drugs should not be attempted during the acute disease, since they may confuse or worsen the clinical picture of patients due to an unexplained cross-reactivity between the drugs. The prognosis is generally worse in the elderly, while the recovery is usually faster and complete in children. For many years, the treatment of DRESS has been based on the use of systemic corticosteroids (2 mg/kg/day) with marked improvement of symptoms and laboratory parameters [8]. After recognizing the clinical and biochemical improvement of the patient, systemic corticosteroids should be tailed off slowly over a period of 6–8 weeks in order to prevent recurrences of the symptoms [12]. If symptoms get worse despite the use of oral corticosteroids, other options used in case series are pulsed methylprednisolone (30 mg/kg intravenously for 3 days), intravenous immunoglobulin (IVIG), and plasmapheresis, or a combination of these therapies [12, 13].

## 4. Conclusion

Due to the significant mortality and morbidity associated with DRESS syndrome, the clinicians should be aware of the possibility of the severe hypersensitivity reaction specially when starting any new antiepileptic medication. When patients present with skin rash and systemic abnormalities after recent changes in medications, clinicians should consider DRESS syndrome as a possible differential diagnosis and use more aggressive therapy if removal of the offending drug does not result in clinical improvement of the patient. Further study of potential pharmacological therapies is required, given the significant morbidity and mortality associated with DRESS syndrome.

## Figures and Tables

**Table 1:** Drug groups commonly associated with drug reaction with eosinophilia and systemic symptoms syndrome.

Drug groups	Specific examples
Anticonvulsants	Phenytoin, carbamazepine, phenobarbital, lamotrigine, valproate
Antidepressants	Desipramine, amitriptyline, fluoxetine
Sulfonamides/sulfones	Dapsone, sulfasalazine, trimethoprim-sulfamethoxazole
Anti-inflammatory drugs	Piroxicam, naproxen, diclofenac, sulindac, ibuprofen
Anti-infective drugs	Abacavir, nevirapine, linezolid, doxycycline, nitrofurantoin
Angiotensin-converting enzyme inhibitors	Captopril, enalapril
Beta-blockers	Atenolol, celiprolol

## Data Availability

The data used for this case study are available from the corresponding author upon reasonable request.
